# Enantioseparation and Determination of Mephedrone and Its Metabolites by Capillary Electrophoresis Using Cyclodextrins as Chiral Selectors

**DOI:** 10.3390/molecules25122879

**Published:** 2020-06-23

**Authors:** Pavel Řezanka, Denisa Macková, Radek Jurok, Michal Himl, Martin Kuchař

**Affiliations:** 1 Department of Analytical Chemistry, Faculty of Chemical Engineering, University of Chemistry and Technology, Prague, Technická 5, 166 28 Prague 6, Czech Republic; Denisa174@gmail.com; 2 Department of Chemistry of Natural Compounds, Forensic Laboratory of Biologically Active Substances, Faculty of Food and Biochemical Technology, University of Chemistry and Technology, Prague, Technická 5, 166 28 Prague 6, Czech Republic; Radek.Jurok@vscht.cz (R.J.); Martin.Kuchar@vscht.cz (M.K.); 3 Department of Organic Chemistry, Faculty of Chemical Technology, University of Chemistry and Technology, Prague, Technická 5, 166 28 Prague 6, Czech Republic; Michal.Himl@vscht.cz

**Keywords:** capillary electrophoresis, chiral separation, cyclodextrin, mephedrone, metabolites

## Abstract

Mephedrone, a psychoactive compound derived from cathinone, is widely used as a designer drug. The determination of mephedrone and its metabolites is important for understanding its possible use in medicine. In this work, a method of capillary electrophoresis for the chiral separation of mephedrone and its metabolites was developed. Carboxymethylated β-cyclodextrin was selected as the most effective chiral selector from seven tested cyclodextrin derivates. Based on the simplex method, the optimal composition of the background electrolyte was determined: at pH 2.75 and 7.5 mmol·L^−1^ carboxymethylated β-cyclodextrin the highest total resolution of a mixture of analytes was achieved. For mephedrone and its metabolites, calibration curves were constructed in a calibration range from 0.2 to 5 mmol·L^−1^; limits of detection, limits of quantification, precision, and repeatability were calculated, and according to Mandel’s fitting test, the linear calibration ranges were determined.

## 1. Introduction

Mephedrone, a psychostimulant drug first synthesized in the 1920s, is classified as a synthetic cathinone [1]. According to the European Monitoring Centre for Drugs and Drug Addiction [2], synthetic cathinones, together with synthetic cannabinoids, are the most abundant substances in the group of so-called new psychoactive substances. In most cases, these drugs have stimulant entactogenic effects; however, the biological activity of such substances is difficult to predict and some of these artificially prepared substances have been shown to have potential therapeutic uses [3], for example, bupropion is used to treat depression and smoking cessation [4] and diethylpropion is prescribed as an anti-obesity drug [5]. On the other hand, pyrovalerone has been prescribed for the treatment of chronic obesity and lethargy but has been withdrawn for its abuse by patients [4,6]. These new psychoactive substances can also mimic prescription therapeutic psychoactive drugs; for example, phenmetrazine, modafinil, and methylphenidate, and hence are more available even for healthy people because they are available on the Internet [7]. So, an intensive study of these compounds is needed.

The most commonly used techniques for isolating new psychoactive substances from biological samples are liquid-to-liquid extraction (LLE) and solid-phase extraction (SPE) [8]. LLE is a simple method, but its problems are easy contamination and matrix influence. On the contrary, the SPE is very selective but time-consuming [9]. A popular method is the QuEChERS method (quick, easy, cheap, effective, rugged, and safe) modified in 2012 for the extraction of psychoactive substances from biological samples [10].

Simple colorimetric tests are used for rapid orientation detection of drugs. However, the identification is very indicative, detecting only the presence of certain structural motifs [11]. However, immunological methods that are commercially available are more commonly used for rapid drug detection. However, there is no sufficiently effective immunoassay on the market for the detection of cathinones that does not show cross-reactivity between analogs [9,12,13].

Of the advanced chromatographic techniques, gas and liquid chromatography with a mass spectrometer (GC-MS and LC-MS) are the most suitable for the analysis of synthetic cathinones. In general, the GC-MS technique is used more for drug analysis, mainly due to shorter elution times [14,15,16,17,18,19]. Chiral separation is most often performed in an achiral environment after the previous conversion of enantiomers to diastereoisomers [20], which, however, brings complications for the quantification itself [21]. It should be also noted that cathinones are thermodegradable substances and due to the thermal conditions necessary for separation by GC, their partial decomposition occurs [5]. Therefore, LC-MS [22,23,24] and LC-UV [25,26] using a chiral stationary or mobile phase is often used. Rapid separation of cathinones is also possible by supercritical fluid chromatography (SFC) [27,28]. Carnes et al. compared the separation of cathinones by ultrahigh efficiency SFC (UHPSFC), ultrahigh efficiency LC (UHPLC) with a non-polar column, UHPLC with a hydrophilic column, and GC with a weakly polar column. The combination of GC and UHPSFC, which is the fastest of the tested methods, seems to be ideal [29].

The first enantioseparation of cathinone derivatives by capillary electrophoresis (CE) was performed by Mohr et al. in 2012 using sulfated β-cyclodextrin as a chiral selector [30]. As with HPLC, CE-UV and CE-MS techniques are used [31,32,33]. For example, to study the incorporation of cathinones into hair, a CE method was developed in which extraction from hair at an elevated temperature and pressure into ammonium hydroxide solution was first performed. Subsequently, the extract was concentrated on a solid phase that was part of the CE capillary (inline coupling of SPE-CE), and analysis was performed using various cyclodextrins as chiral selectors [34].

Mephedrone is referred to as a catecholamine reuptake inhibitor [35,36,37], but it also positively affects the release of catecholamines into the synaptic cleft [13,38,39,40,41]. Therefore, it seems to have a mixed effect [42,43,44]. In both cases, the result is an increase in the concentration of catecholamines in the synaptic cleft, resulting in the typical action of psychostimulants. Acute intoxication with mephedrone, usually in combination with other drugs and alcohol, has already resulted in hundreds of deaths across Europe [9].

**Figure 1:**
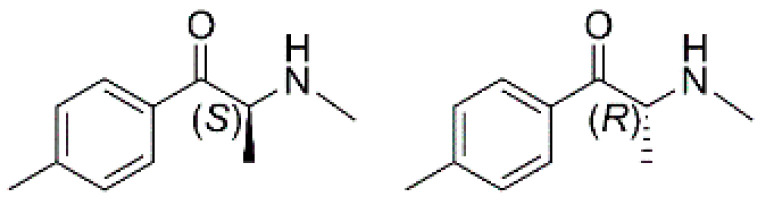
Structures of (*S*)- and (*R*)-mephedrone.

Mephedrone has a chiral center on the α carbon and exists in two enantiomeric forms (Figure 1). Both enantiomers have a similar affinity for dopaminergic transporters, but (*S*)-mephedrone is about 50 times more potent as a serotonergic mediator. Nevertheless, the (*R*)-enantiomer is primarily responsible for the euphoric effects [45].

**Figure 2:**
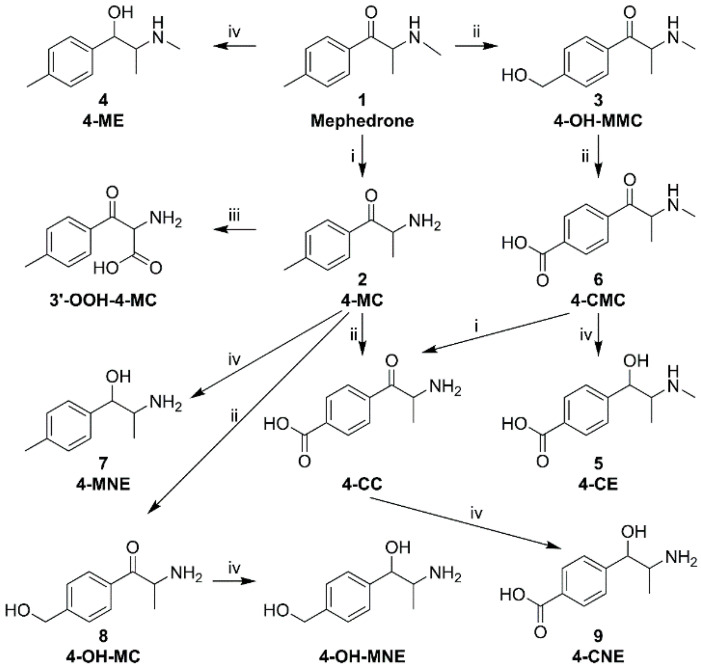
Scheme of phase I biotransformation of mephedrone: (i) oxidative *N*-demethylation, (ii) oxidation of the 4-methyl group, (iii) ω-oxidation at the 3′ position, and (iv) reduction of the carbonyl group.

In phase I biotransformation, mephedrone can undergo several reactions: (i) oxidative *N*-demethylation, (ii) oxidation of the 4-methyl group, (iii) ω-oxidation at the 3′ position, and (iv) reduction of the carbonyl group (Figure 2) [46].

The major metabolites of mephedrone (**1**; 4-methyl methcathinone) are 4-methylcathinone (**2**; 4-MC) and 4-hydroxymethyl methcathinone (**3**; 4-OH-MMC). These, together with 4-methylephedrine (**4**; 4-ME), 4-carboxymethylcathinone (**6**; 4-CMC), 4-methylnorephedrine (**7**; 4-MNE), and normephedrone-ω-carboxylic acid (3′-OOH-4-MC), were identified in the urine of users by ultra-high-performance liquid chromatography‒mass spectrometry (UHPLC-MS) [47]. Other metabolites, namely 4-carboxyephedrine (**5**; 4-CE), 4-MNE (**7**), 4-hydroxymethylcathinone (**8**; 4-OH-MC) [17], 4-carboxynorephedrine (**9**; 4-CNE), 4-carboxycathinone (4-CC), and 4-hydroxymethylnorephedrine (4-OH-MNE) [46] were found in the urine of rats by GC-MS and LC-MS.

The aim of this work was to find the optimal conditions for the separation of mephedrone and its metabolites by capillary electrophoresis (for details about this method, see, for example, Bernardo-Bermejo et al. [48]), using cyclodextrins (CDs) [49] as chiral selectors. The optimization of the background electrolyte (BGE) was carried out by using the simplex procedure, which was thoroughly described by Catai and Carrilho [50]. Mephedrone and all its available metabolites, selected on the basis of current knowledge on the biotransformation of mephedrone, are chiral substances that may undergo different metabolic pathways and thus have different biological effects for individual enantiomers.

## 2. Results and Discussion

### 2.1. Cyclodextrin Optimization

**Figure 3:**
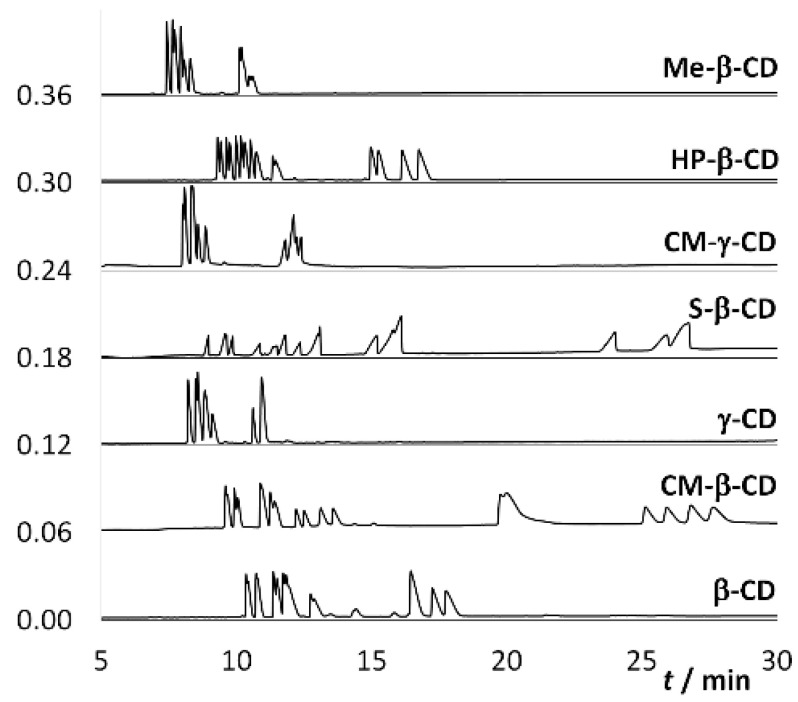
Electropherograms of the analyzed mixture of the studied analytes 1–9 (Figure 2) obtained at different CDs used; fused-silica capillary: od/id = 375/75 μm, total/effective length: 58.5/50.0 cm; BGE: 50 mmol·L^−1^ phosphate buffer, pH 2.5, 10 mmol·L^−1^ CD; voltage: 20 kV (−20 kV for S-β-CD); temperature: 25 °C.

**Figure 4:**
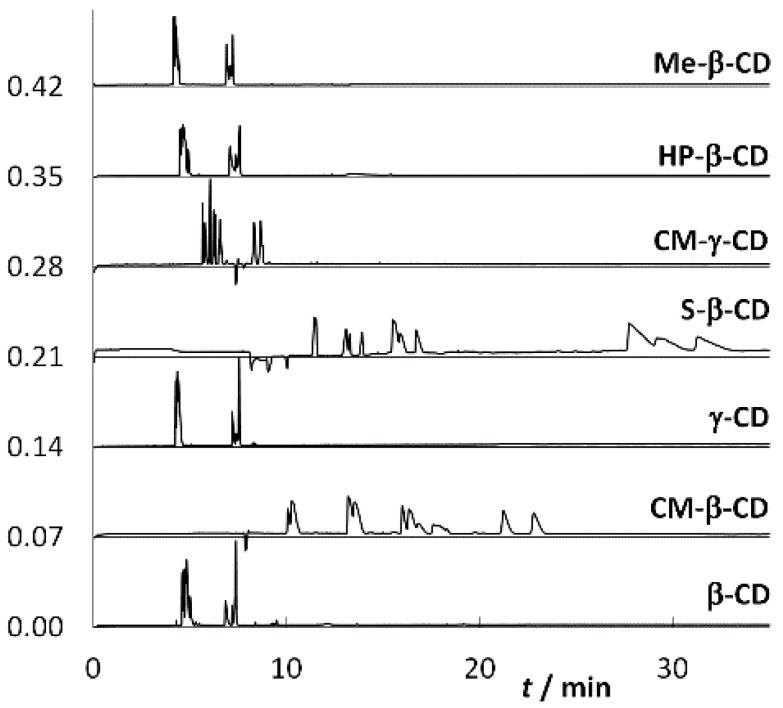
Electropherograms of the analyzed mixture of the studied analytes 1–9 (Figure 2) obtained at different CDs used; fused-silica capillary: od/id = 375/75 μm, total/effective length: 58.5/50.0 cm; BGE: 50 mmol·L^−1^ acetate buffer, pH 5, 10 mmol·L^−1^ CD; voltage: 20 kV; temperature: 25 °C.

**Figure 5:**
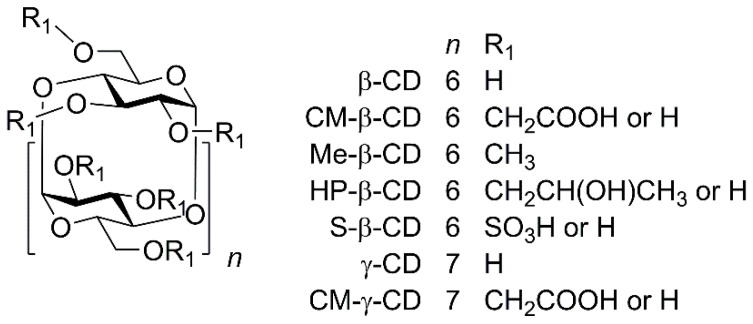
Structures of CDs derivatives used as chiral selectors.

The first optimization step was to select a suitable CD. Based on our previous experience with CDs [51], analyses were performed in the presence of CDs at a concentration of 10 mmol·L^−1^, either in a 50 mmol·L^−1^ phosphate buffer at pH 2.5 (Figure 3) or in a 50 mmol·L^−1^ acetate buffer at pH 5 (Figure 4). From the measured electropherograms, it was concluded that the separation proceeds better in the phosphate buffer at a lower pH value. From the selected CDs (Figure 5), i.e., β-CD, carboxymethylated β-cyclodextrin (CM-β-CD), heptakis(2,3,6-tri-*O*-methyl)-β-cyclodextrin (Me-β-CD), 2-hydroxypropylated β-cyclodextrin (HP-β-CD), sulfated β-cyclodextrin (S-β-CD), γ-CD, and carboxymethylated γ-cyclodextrin (CM-γ-CD), CM-β-CD, which is often used for the separation of basic nitrogenous substances [52,53], seems to be the most suitable. This may be due to the interaction of the carboxyl group of CM-β-CD with the NH group of the analyte and also due to the appropriate size of the β-CD cavity. The inappropriateness of S-β-CD may be due to too many sulfate groups (7–11) compared to three carboxylic groups of CM-β-CD, see Section 3.1. In its presence, 18 diastereomers were separated within 30 min, so it was used in all the other work. The advantage of CM-β-CD over β-CD is its higher solubility in water (50 mg·mL^−1^ compared to 19 mg·mL^−1^). Thus, in the search for optimal separation conditions, it is possible to achieve higher concentrations if necessary.

### 2.2. BGE Optimization

The enantioselective separation of ionic analytes with ionic CDs is controlled by at least two mechanisms. The first is the interaction of the hydrophobic cavity of the CD derivative with the analyte; the second is the electrostatic interaction between the ionic groups of the CD derivative and the analyte [54]. The approximate value of the dissociation constant p*K*_a_ CM-β-CD is 3.0–3.5 [55]. Cathinones are basic drugs and have high values of dissociation constants; for example, the p*K*_a_ of mephedrone is 8.77 [56], for methylated cathinones it is 8.4–9.5 [57], for phenylethylamine it is 10.3 [58], and for methamphetamine it is 9.9 [59]. In addition to pH, the separation is also affected by the concentration of cyclodextrin, which affects the amount of complexed analyte and thus affects its overall electrophoretic mobility [49]. The separation efficiency can be increased at higher voltages, but this also increases the temperature difference in the middle and at the edge of the capillary, which leads to a decrease in efficiency. Based on our experience with a similar BGE [51], we have chosen a voltage (see Section 3.3) such that the current is around 50 μA. In addition to voltage, the current also depends on the ionic strength of the BGE. Again, we used our previous experience here, and in most cases used a buffer with a concentration of 50 mmol·L^−1^.

**Figure 6:**
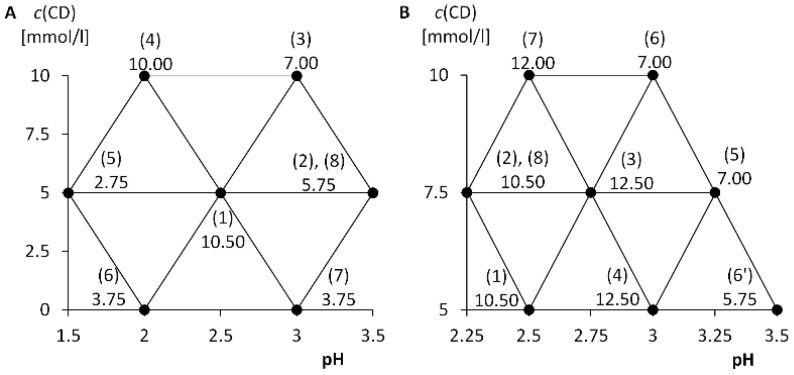
Simplex procedures in optimization of separation conditions; (**A**) full-simplex; (**B**) half-simplex; fused-silica capillary: od/id = 375/75 μm, total/effective length: 58.5/50.0 cm; BGE: 50 mmol·L^−1^ phosphate buffer (46.6 mmol·L^−1^ HCl at pH 1.5), variable CM-β-CD concentration, variable pH; voltage: 20 kV (10 kV for pH 1.5); temperature: 25 °C.

Thus, the optimization step was to find a suitable concentration of CM-β-CD and a suitable pH of BGE. In order to monitor both parameters simultaneously, the simplex method was used to find the optimal conditions [60]. The first step was to construct an initial simplex. The first two points correspond to the conditions: (1) pH 2.5 and 5 mmolv·L^−1^ CM-β-CD; (2) pH 3.5 and 5 mmol·L^−1^ CM-β-CD (Figure 6A). The third point was calculated on the basis of the properties of an equilateral triangle, i.e., pH_3_ = 2.5 + 0.5·1 = 3, where 2.5 corresponds to the first point and value 1 to the length of the simplex edge on the pH axis, and *c*_3_ = 5 + √3⁄2·5.774 = 10 mmol·L^−1^, where 5 is the initial concentration and 5.774 is the length of the simplex edge corresponding to the *c*-axis, which has been chosen so that the CM-β-CD concentration is an integer.

A mixture of analytes 1–9 (Figure 2) with a concentration of 1 mmol·L^−1^ was measured twice under each pair of conditions. Based on the resolution of the peaks of the individual enantiomers, the average criterion *R*_kr_ was calculated (see above), which served as a compared response for the simplex procedure. Simplex followed the rules of the equilateral simplex procedure. After calculating the coordinates of point (8), the simplex looped. Thus, the chiral separation proceeded best under the initial conditions chosen. The simplex procedure is summarized in Figure 6A.

A half-simplex was designed to more accurately determine the optimal chiral separation conditions (Figure 6B). The first point corresponds to the first determined optimal conditions: (1) pH 2.5 and 5 mmol·L^−1^ CM-β-CD. The other two points correspond to: (2) pH_2_ = 2.5 − 0.5·0.5 = 2.25 and *c*_2_ = 5 + √3⁄2·2.887 = 7.5; (3) pH_3_ = 2.5 + 0.5·0.5 = 2.75 and *c*_3_ = *c*_2_, where the values 0.5 and 2.887 correspond to half the sizes of the edges of the first simplex.

**Figure 7:**
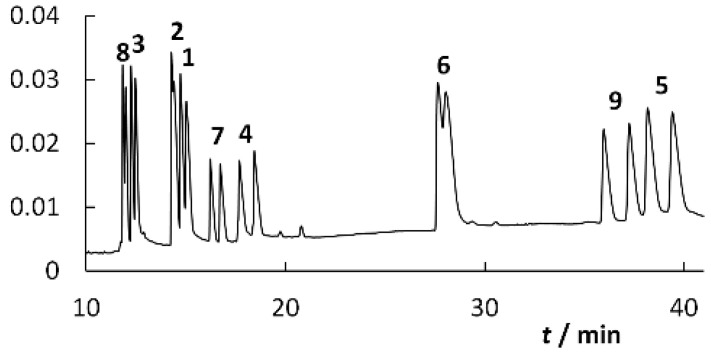
Electropherogram of the analyzed mixture of the studied analytes 1–9 (Figure 2) obtained; fused-silica capillary: od/id = 375/75 μm, total/effective length: 58.5/50.0 cm; BGE: 50 mmol·L^−1^ phosphate buffer, pH 2.75, 7.5 mmol·L^−1^ CM-β-CD; voltage: 20 kV; temperature: 25 °C.

Furthermore, the simplex proceeded according to the rules of the equilateral simplex procedure. Since points (3) and (4) provide the same response, *R*_kr_ = 12.5, it is possible in a situation where the simplex forms points (3), (4), and (5), to proceed in two directions, namely to point (6) and point (6′), which gives a small response. Points (3) and (4) also provide the maximum value of the criterion *R*_kr_. Point (3) was chosen as the point meeting the optimal conditions, as in the second case the analysis was extended to more than 100 min. The simplex procedure is summarized in Figure 6B. The resulting optimum was found at pH 2.75 and 7.5 mmol·L^−1^ CM-β-CD (Figure 7).

At pH 2.75, approximately 65–85% of CM-β-CD is in a protonated form. At a low pH, most of the silanol groups on the capillary wall are protonated, leading to low electroosmotic flow, and CM-β-CD carries an approximately one-fold negative charge. At pH 2.75, the amine groups of all analytes are practically 100% protonated. Metabolites 5, 6, and 9 carry a carboxyl group on the aromatic nucleus in the para position. Upon approximation with benzoic acid, whose p*K*_a_ is 4.21, it can be assumed that at pH 2.75 the carboxyl group is 4% deprotonated. Thus, it is clear that pH plays an important role in the separation. A lower or higher pH than 2.75 affects the degree of deprotonation of the used CM-β-CD, and thus the separation efficiency.

The concentration of CM-β-CD is also an important factor as it affects how much analyte will be complexed with CD. For a given racemic mixture, there is an optimal concentration at which the individual enantiomers will be separated as best as possible [49,61]. This concentration depends on the stability constants of the individual enantiomers and the given chiral selector. In the case of separation of a mixture of racemic substances, as in our case, it is necessary to choose a compromise between these concentrations.

### 2.3. Calibration

Calibration solutions were measured under the optimized BGE composition, namely at 7.5 mmol·L^−1^ CM-β-CD and pH 2.75. Reduced peak areas (area/migration time) were subjected to the Dean–Dixon test to exclude outliers, which is adapted to small datasets with unknown distributions.

Furthermore, the regression parameters of the calibration curves were calculated. Based on the test of the significance of the parameter, we assessed whether it is possible to set the intercept *a* in the regression equation *y* = *bx* + *a*. In the case of analytes 1, 2, 5, and 7, the size of the intercept is insignificant according to the test. Subsequently, the confidence intervals for the intercept *a* and the slope *b* of all calibration equations were calculated. If the significance test of the parameter did not allow for ignoring the intercept, the number of degrees of freedom for calculating *t*_crit_ was equal to *n* − 2, and in the case of a zero intercept *n* − 1. Ignoring the intercept improved the values of the coefficient of determination *R*^2^ and thus allowed us to reduce the confidence intervals (Table 1).

According to Mandel’s test, linear concentration ranges were determined and subsequently the LOD and LOQ of individual analytes were calculated (Table 2). Precision for all studied analytes was <4.9% and repeatability <4.5%.

## 3. Materials and Methods 

### 3.1. Chemicals and Materials

The following substances were used: ortho-phosphoric acid (50%), β-cyclodextrin (β-CD, 97%), carboxymethylated β-cyclodextrin sodium salt (CM-β-CD, average degree of substitution ~3, 95%), 2-hydroxypropylated β-cyclodextrin (HP-β-CD, average degree of substitution ~0.5–1.3, 95%), sulfated β-cyclodextrin (S-β-CD, average degree of substitution ~7–11, 95%), acetic acid (99%), thiourea (99%) (all Sigma-Aldrich, Praha, Czech Republic), ultrapure water (Milli-Q grade, Millipore, Molsheim, France), 1 mol·L^−1^ sodium hydroxide (Tripur, Merck, Darmstadt, Germany), hydrochloric acid (30%)(Suprapur, Merck, Darmstadt, Germany), γ-cyclodextrin (γ-CD, 97%, FUJIFILM Wako, Richmond, VA, USA), carboxymethylated γ-cyclodextrin sodium salt (CM-γ-CD, average degree of substitution ~3–6, 95%, AraChem, Kuala Lumpur, Malaysia), and heptakis(2,3,6-tri-*O*-methyl)-β-cyclodextrin (Me-β-CD, 98%, Fluka, Munich, Germany). Psychoactive amines 1–9 (Figure 2) in the form of hydrochloride salts were prepared previously [46]. The purity of the salts studied was determined by UHPLC/MS Agilent 6460 (Waldbronn, Germany) and was higher than 95%.

### 3.2. Equipment

CE separations were performed with an Agilent CE instrument 7100 (Agilent 3D HPCE, Waldbronn, Germany) equipped with a UV-Vis diode-array detector. A bare fused-silica capillary of 375/75 μm od/id and 58.5/50 cm total/effective length was obtained from Polymicro Technologies (Phoenix, AZ, USA) was used.

### 3.3. CE Conditions

The BGE used for the optimizing of the chiral selector consisted of 50 mmol·L^−1^ sodium phosphate buffer, pH 2.5 (50 mmol·L^−1^ orthophosphoric acid adjusted to appropriate pH with 1 mol·L^−1^ NaOH) or acetate buffer, pH 5.0 (50 mmol·L^−1^ acetic acid adjusted to appropriate pH with 1 mol·L^−1^ NaOH), and 10 mmol·L^−1^ of the studied CDs (β-CD, γ-CD, CM-β-CD, CM-γ-CD, HP-β-CD, 2-Me-β-CD, or S-β-CD). The background electrolyte used for the optimizing of the chiral selector concentration and pH consisted of 46.6 mmol·L^−1^ hydrochloric acid (pH 1.5) or 50 mmol·L^−1^ phosphate buffer (pH 2; 2.25; 2.5; 2.75; 3; 3.25; 3.5) and 0–10 mmol·L^−1^ of CM-β-CD.

A new fused-silica capillary was first rinsed with 1 mol·L^−1^ NaOH for 30 min, then with H_2_O for 30 min. Between the runs, the capillary was rinsed at 99.4 kPa first with 0.1 mol·L^−1^ NaOH for 2 min, then with H_2_O also for 2 min, and finally with the running buffer again for 2 min (for the capillary washing, a different buffer solution than for the subsequent analysis was used). The analytes were injected hydrodynamically at a pressure of 1.5 kPa for 5 s. Separations were performed at 10 kV, 20 kV (anode at the injection capillary end), or −20 kV with a voltage ramp time of 12 s. Detection was carried out at 207 nm during the optimization step and 258 nm for analytes 1, 2, 3, and 6; 236 nm for analyte 5; and 214 nm for analytes 4 and 7 (i.e., at wavelengths corresponding to the absorption maxima) during the calibration step. The capillary was thermostated at 25 °C during the analyses.

### 3.4. Sample Preparation

Individual analytes were dissolved in water at a 20 mmol·L^−1^ concentration. For optimizing the chiral selector, the analyte solutions were further mixed and diluted with water to a final concentration of 1 mmol·L^−1^. For calibration, seven different mixtures of analytes 1–7 (Figure 2) at concentration ranges from 0.2 to 5 mmol·L^−1^ (the sum of all analytes’ concentrations was 10 mmol·L^−1^) were prepared and each mixture was analyzed five times. 

### 3.5. Simplex Method

For optimizing the BGE composition, the two-dimensional simplex method was used [60]. The optimizing parameters were CM-β-CD concentration and electrolyte pH. As an evaluation criterion, *R*_cr_ was introduced and calculated as follows. For resolution between two neighborhood peaks *i* and *j*, if (*R*_s,ij_) < 0.5 then *R*_cr,ij_ = 0, if 0.5 < *R*_s,ij_ < 1.5 then *R*_cr,ij_ = 0.5, and if *R*_s,ij_ > 1.5 then *R*_cr,ij_ = 1. The sum of *R*_cr,ij_ = *R*_cr_.

## 4. Conclusions

In this work, the composition of the basic electrolyte for the chiral separation of mephedrone and its selected metabolites was optimized. A total of seven cyclodextrin derivatives were selected as potential chiral selectors. From the seven tested cyclodextrins, carboxymethylated β-cyclodextrin was selected as the most suitable. Cyclodextrin concentration and pH were determined based on the simplex method for two parameters. The optimized pH was found to be 2.75 and the concentration of carboxymethylated β-cyclodextrin was 7.5 mmol·L^−1^. A total of nine analytes were present in the mixture, and 18 distinguishable peaks were found. Thus, all analytes were separated.

Furthermore, the data for the construction of calibration dependences were measured. According to the Dean‒Dixon test, outliers were excluded, and regression parameters of calibration lines were calculated. Based on the test of significance of the parameter, the intercept on the *y*-axis was neglected in some cases. Finally, linear concentration ranges according to Mandel’s test and detection limits, limits of determination, precision, and repeatability were determined.

## Figures and Tables

**Table 1:** Calibration dependences of separation of analytes 1–7 (Figure 2) measured under optimal conditions, where (*a* ± *L* (*a*)) is the intercept and (*b* ± *L* (*b*)) is the slope with a confidence interval at the significance level *α* = 0.05; *R*^2^ is the coefficient of determination.

Analyte	*a*		*L*(*a*)	*b*		*L*(*b*)	*R* ^2^
				mmol·L^−1^		mmol·L^−1^	
1	0			32.60	±	0.27	0.991
2	0			27.76	±	0.16	0.995
3	−5.47	±	1.31	23.54	±	1.14	0.807
4	−5.07	±	0.65	19.68	±	0.29	0.967
5	0			26.14	±	0.21	0.989
6	−3.55	±	1.07	36.01	±	0.83	0.959
7	0			21.04	±	0.20	0.991

**Table 2:** LOD and LOQ with a linear concentration range of calibration dependences of individual analytes 1–7 (Figure 2) according to Mandel’s test.

Analyte	LOD mmol·L^−1^	Linear Concentration Range		
		LOQ	mmol·L^−1^	
1	0.35	1.17	−	5.00
2	0.28	0.92	−	5.00
3	0.61	2.02	−	2.20
4	0.53	1.76	−	5.00
5	0.46	1.53	−	5.00
6	0.31	1.04	−	2.20
7	0.21	0.71	−	2.20
